# Supplementary material for: Morin Hydrate Reverses Cisplatin Resistance by Impairing PARP1/HMGB1-Dependent Autophagy in Hepatocellular Carcinoma
Source: Cancers (Basel). 2019 Jul 15;11(7):986. doi: 10.3390/cancers11070986 (PMC6678360; doi:10.3390/cancers11070986)
Supplement: Supplementary file 1 [file cancers-11-00986-s001.pdf]

Supplementary Materials

Morin Hydrate Reverses Cisplatin Resistance by Impairing PARP1/HMGB1-Dependent Autophagy in Hepatocellular Carcinoma

Mahendra Pal Singh, Hee Jun Cho, Jong-Tae Kim, Kyoung Eun Baek, Hee Gu Lee and Sun Chul Kang

Table S1. List of antibodies for Western blot analysis.

Antibodies	Dilution Used	Source	Catalog No.
β-actin	1:1000	Santa Cruz Biotechnology	sc-1616
Lamin B	1:1000	Santa Cruz Biotechnology	sc-6217
ERCC1	1:1000	Santa Cruz Biotechnology	sc-17809
ABCB-1	1:1000	Santa Cruz Biotechnology	sc-104019
HMGB-1	1:1000	Cell signalling	#3935
PARP-1	1:1000	Santa Cruz Biotechnology	sc-7150
PAR	1:1000	Calbiochem	AM80
SOD-1	1:1000	Santa Cruz Biotechnology	sc-11407
SOD-2	1:1000	Santa Cruz Biotechnology	sc-30080
Catalase	1:1000	Santa Cruz Biotechnology	sc-50508
Glutathione reductase	1:1000	Santa Cruz Biotechnology	sc-32886
JNK	1:1000	Santa Cruz Biotechnology	sc-571
p-JNK	1:1000	Santa Cruz Biotechnology	sc-12882
Bcl-2	1:1000	Cell signalling	#2876
p38	1:1000	Santa Cruz Biotechnology	sc-535
p-P38	1:1000	Santa Cruz Biotechnology	sc-17853-R
p53	1:1000	Santa Cruz Biotechnology	sc-6243
PUMA	1:1000	Santa Cruz Biotechnology	sc-28226
Cyt C	1:1000	Cell signalling	#4272
Casp-9	1:1000	Santa Cruz Biotechnology	sc-7885
Casp-3	1:1000	Santa Cruz Biotechnology	sc-7148
H2A.X	1:1000	Santa Cruz Biotechnology	sc-517336
LC3I/II	1:500	Abcam	ab58610
ATG-5	1:1000	Cell Signalling	#12994
ATG-7	1:1000	Cell Signalling	#8558
BECN-1	1:1000	Cell Signalling	#3495
p-62	1:1000	Cell Signalling	#5114
PI3-Kinase p85α	1:1000	Santa Cruz Biotechnology	sc- 1637

Table S2. Sequences of RT-PCR oligonucleotide primers.

Gene	Direction	Sequences
Cyt c	**Forward**	5′- CAA CAC CTC TCA CAT CTT AC-3′
	**Reverse**	5′-TCC CCA ATC AAA TAC ACA GTT-3′
Casp-3	**Forward**	5′- GTG CTA CAA TGC CCC TGG AT-3′
	**Reverse**	5′- GCC CAT TCA TTT ATT GCT TTC C-3′
PARP-1	**Forward**	5′-TTC ACA TAT CAG CAA GTT ACC-3′
	**Reverse**	5′-CCT GAG CAA TAT CAT AGA CAA T-3′
BECN-1	**Forward**	5′-TGG CAC AAT CAA TAA CTT CA-3′
	**Reverse**	5′-TAA GGA ACA AGT CGG TAT CT-3′
LC3II	**Forward**	5′-GAG GTG TAT GAG AGT GAG AA-3′
	**Reverse**	5′-CTG TGA TTG GAT GAA CTG AT-3′
β-actin	**Forward**	5′-AAC TAC CTT CAA CTC CAT CA-3′
	**Reverse**	5′-GAG CAA TGA TCT TGA TCT TCA-3′

Table S3. Sequences of SiRNA for ATG5.

Gene	Direction	Sequences
Atg5	**Sense**	5′- ACGCUAAAAGGCUUACAGUAUCAGA-3′
	**Antisense**	5′-UCUGAUACUGUAAGCCUUUUAGCGUAC-3′

© 2019 by the authors. Licensee MDPI, Basel, Switzerland. This article is an open access article distributed under the terms and conditions of the Creative Commons Attribution (CC BY) license (http://creativecommons.org/licenses/by/4.0/).
